# Survival, Growth and Reproduction of Non-Native Nile Tilapia II: Fundamental Niche Projections and Invasion Potential in the Northern Gulf of Mexico

**DOI:** 10.1371/journal.pone.0041580

**Published:** 2012-07-27

**Authors:** Michael R. Lowe, Wei Wu, Mark S. Peterson, Nancy J. Brown-Peterson, William T. Slack, Pamela J. Schofield

**Affiliations:** 1 Department of Coastal Sciences, University of Southern Mississippi, Ocean Springs, Mississippi, United States of America; 2 United States Army Engineer Research and Development Center, Waterways Experiment Station, Vicksburg, Mississippi, United States of America; 3 United States Geological Survey, Southeast Ecological Science Center, Gainesville, Florida, United States of America; Technical University of Denmark, Denmark

## Abstract

Understanding the fundamental niche of invasive species facilitates our ability to predict both dispersal patterns and invasion success and therefore provides the basis for better-informed conservation and management policies. Here we focus on Nile tilapia (*Oreochromis niloticus* Linnaeus, 1758), one of the most widely cultured fish worldwide and a species that has escaped local aquaculture facilities to become established in a coastal-draining river in Mississippi (northern Gulf of Mexico). Using empirical physiological data, logistic regression models were developed to predict the probabilities of Nile tilapia survival, growth, and reproduction at different combinations of temperature (14 and 30°C) and salinity (0–60, by increments of 10). These predictive models were combined with kriged seasonal salinity data derived from multiple long-term data sets to project the species' fundamental niche in Mississippi coastal waters during normal salinity years (averaged across all years) and salinity patterns in extremely wet and dry years (which might emerge more frequently under scenarios of climate change). The derived fundamental niche projections showed that during the summer, Nile tilapia is capable of surviving throughout Mississippi's coastal waters but growth and reproduction were limited to river mouths (or upriver). Overwinter survival was also limited to river mouths. The areas where Nile tilapia could survive, grow, and reproduce increased during extremely wet years (2–368%) and decreased during extremely dry years (86–92%) in the summer with a similar pattern holding for overwinter survival. These results indicate that Nile tilapia is capable of 1) using saline waters to gain access to other watersheds throughout the region and 2) establishing populations in nearshore, low-salinity waters, particularly in the western portion of coastal Mississippi.

## Introduction

Aquaculture is the fastest growing food-production sector in the world and is viewed as a viable solution to global nutritional deficiencies and poverty [1], [2]. Given the declining status of wild fish stocks [3], aquaculture may one day surpass capture fisheries in terms of food-fish production [2]. Despite the economic contributions of aquaculture and potential for mitigating environmental impacts [4], aquaculture does not operate in a vacuum [5] and the same traits that make species desirable for production also lend to their potential as an invasive species [6], [7]. After habitat modification, invasive species are among the largest threats to freshwater and marine fish biodiversity [8], [9]. Despite a growing understanding of the consequences of biological invasions [10]–[13] the number of invasive species [14], [15] resulting from aquaculture suggests that there is a trade-off that favors economic benefit over the potential impacts of culturing non-native species [16].

Nile tilapia (*Oreochromis niloticus* Linnaeus, 1758), a secondary freshwater teleost native to the Nilo-Sudanian ecoregion of Africa [17], is one of the most widely cultured species globally and has been introduced to at least 85 countries [18], [19]. Nile tilapia is capable of rapid adaptation [20] and shows a wide range of biological responses to environmental conditions [21], [22]. Further, many cultured tilapiine fishes have been genetically enhanced for the purpose of increased production; thus their response to local environmental conditions, once escaped, may be less predictable [23], [24]. Therefore, it is invalid to assume that environmental constraints prevent the establishment and dispersal of this highly cultured species. For example, it was assumed that the escaped Nile tilapia from aquaculture facilities in coastal Mississippi would not survive through a temperate winter. However, Nile tilapia has become established in coastal Mississippi [7], [25] and its eradication may not be feasible [26], [27]. Further, the spread of established Nile tilapia does not appear to be limited by salinity as Schofield et al. [22] found Nile tilapia from Mississippi could survive, grow, and reproduce in elevated salinity during summer although overwinter survival occurred up to a salinity of 10. Given these important demographics, it was hypothesized that not only do the waters of the Mississippi Sound (hereafter Sound) provide suitable habitat for Nile tilapia to complete all facets of their life history, but also act as a ‘salt-bridge’ [28], whereby Nile tilapia can gain access to other freshwater systems by moving through saline waters, potentially facilitating its spread to other freshwater systems throughout the northern Gulf of Mexico (GOM).

The invasion process is defined by a progression of stages (e.g., introduction, establishment, and spread) that are both biologically and environmentally filtered at each step [29], [30]. Once an invasive species becomes established, regional patterns in abiotic variables become the principal driver of spread. The growing effort to model the distribution and potential spread of invasive species following establishment [31]–[34] has highlighted the need for a proactive, predictive approach to invasive species management [35]. Species distribution models (SDM) based on ecological niche theory have emerged as a common approach to predicting species' ranges [36]. Though powerful tools, SDMs are based on observational data (e.g., presence/absence) and tend to under-perform with small sample size [37], [38]. As a result, a more prudent approach is to use the fundamental niche of ‘data poor’ taxa (e.g., recently established invasive species) to mechanistically predict the potential distribution of an organism based on physiological constraints. Models based on the fundamental niche not only help understand the contemporaneous distribution of an organism in the present climate [36], but also allow the prediction of future distributions under climate change scenarios [39].

Current climate predictions from 16 General Circulation Models downscaled to the northern GOM suggest that air temperature will increase from 2.5–5.9°C in summer, and 1.4–4.5°C in winter from 2070–2099 compared to 1961–1990 under the A2 emission scenario in World Climate Research Programme's Coupled Model Intercomparison Project phase 3 multi-model dataset [40]. Concomitantly, regional precipitation is projected to vary between −28% to 32% in the summer and −48% to 18% in winter. While increasing temperature will likely favor the survival, growth, and reproduction of Nile tilapia [41], [42], the uncertainty surrounding precipitation makes predicting the effect of regional salinity on population spread more problematic.

**Figure 1:**
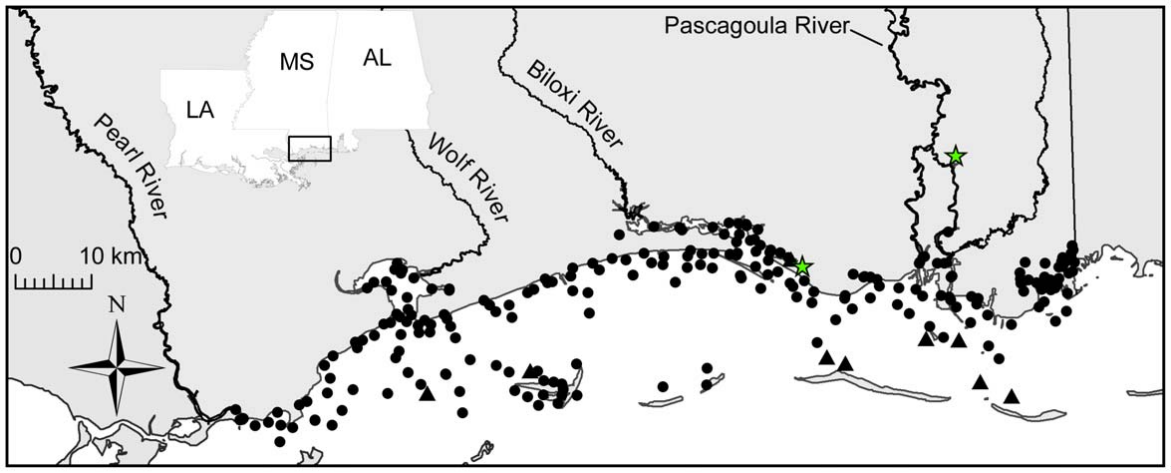
Map of Mississippi Sound and locations of long-term environmental stations used in for salinity projections. Closed circles represent stations that have both summer and winter data while closed triangles only have summer data. The gray stars represent the location of established populations of *Oreochromis niloticus*.

The objectives of this study are to: 1) develop a predictive model of Nile tilapia survival, growth, and reproduction derived from our earlier research [22]; 2) project the species' fundamental niche in the Sound by integrating spatial environmental data (see Figure 1) with the predictive model; 3) project the potential pathways that an established population of Nile tilapia might use to invade other drainages throughout coastal Mississippi and the GOM; and 4) examine how future climate variability may affect the distribution of Nile tilapia. See (Figure S1) for a detailed diagram of our quantitative approach.

## Results

**Figure 2:**
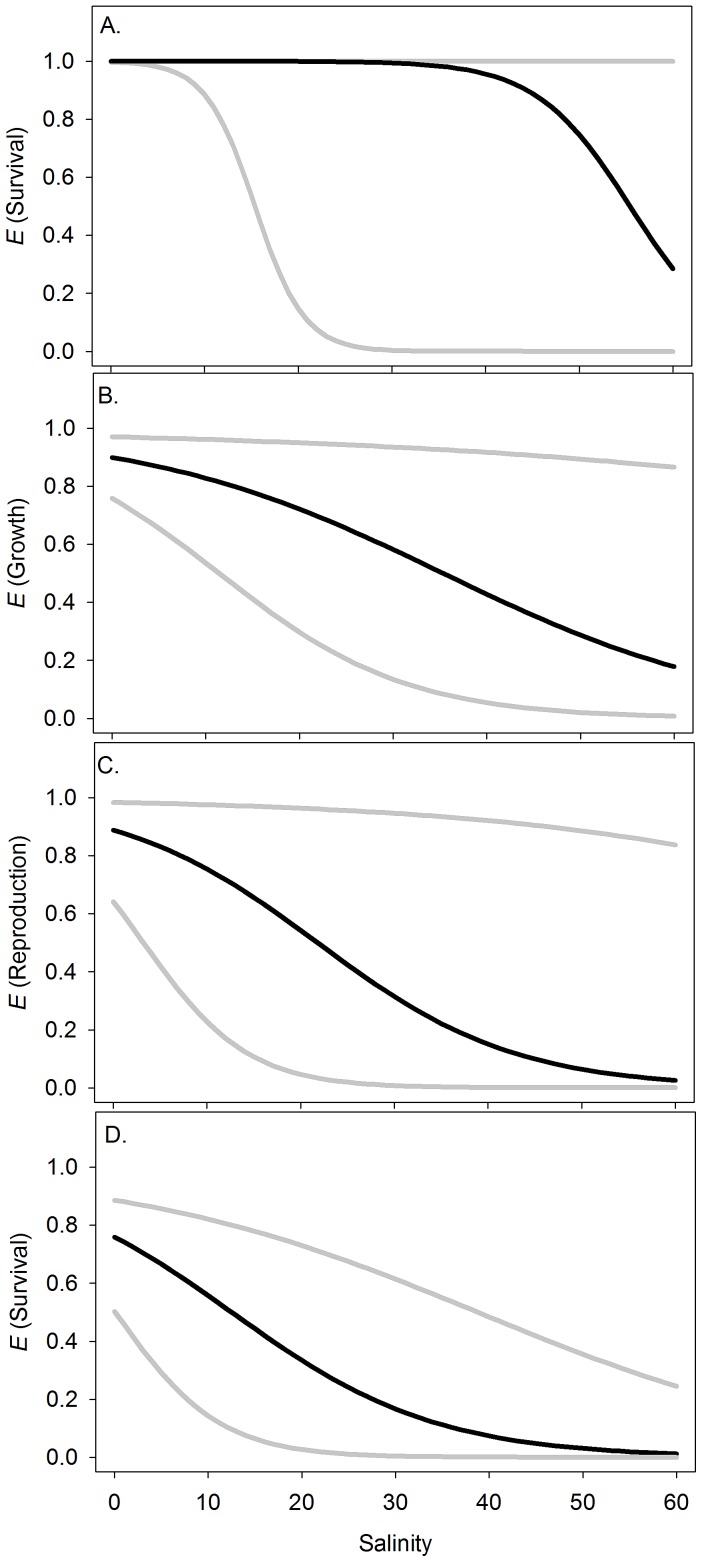
Empirical estimates of the relationships between *Oreochromis niloticus* biological response variables and salinity. A) summer survival, B) summer growth, C) summer reproduction, and D) winter survival. The black line indicates penalized maximum likelihood estimate of the logistic response function (Eq. 2); gray lines are the 95% confidence intervals based on the profile of the penalized likelihoods.

We were able to find a finite estimate for all of our model parameters using the FLR with penalized maximum likelihood estimation. All intercept and slope estimates were significantly different from zero for all models (Table 1; WALD, all *p*-values≤0.008) and the probability of a positive outcome for all biological response (i.e., survival, growth, and reproduction) variables was inversely related to salinity (Table 1; negative slope (b) for all models) in both the summer and winter (Figure 2). Further, all models with salinity as a covariate were statistically more reliable than the intercept only models (Table 1; LRT, all *p*-values≤0.0002) indicating that each model's predictive ability was significantly enhanced by the inclusion of salinity as a covariate.

**Table 1:** Model estimates and associated statistics from Firth-logistic regression.

		Upper	Lower	WALD	LRT	
Parameter	Estimate (SE)	95% CI	95% CI	p-value	p-value	*R* ^2^
Summer						
Survival					38.3	0.76
a	11.08 (3.28)	19.89	5.84	<0.0001	<0.0001	
b	−0.20 (0.063)	−0.11	−0.38	<0.0001		
Growth					13.48	0.61
a	2.19 (0.59)	3.48	1.14	<0.0001	0.0002	
b	−0.062 (0.019)	−0.027	−0.10	<0.0001		
Reproduction					14.18	0.53
a	2.066 (0.88)	4.10	0.58	0.004	<0.0002	
b	−0.095 (0.033)	−0.041	−0.18	0.001		
Winter						
Survival					23.49	0.55
a	0.89 (0.55)	2.06	0.12	0.008	<0.0001	
b	−0.012 (0.031)	−0.053	0.18	<0.0001		

**SE = Standard Error, PML = penalized maximum likelihood, WALD = Wald score, LRT = log ratio test.** ***R^2^*** **= Nagelkerke's R^2^.**

The final semivariogram models fit to the observed salinity data were chosen based on the lowest sum of squared errors among the exponential, spherical, and Gaussian models (Table 2). Normal salinity years were less variable (shown in total sill) than either wet or dry years during both seasons, due to smoothing of extreme salinity values with high (wet years) or low (dry years) river discharge. Micro-scale variability (i.e., nugget effect) in salinity patterns was generally greatest during periods of high river discharge (i.e., winter and wet years) compared to low discharge periods (i.e., summer and dry years). During normal years (Figures S3A and S4A), salinity generally increased from west to east throughout the Sound, was lowest in the bays and estuaries near river mouths and greatest in areas with minimal freshwater input (e.g., offshore barrier islands and saltmarshes of eastern Mississippi) and areas associated with major ship channels (Gulfport and Pascagoula). Further, salinity was greater in the summer (Figure S3A) than in the winter (Figure S4A). While these spatial and seasonal patterns were consistent for both wet (Figures S3B and S4B) and dry years, overall salinity (Figures S3C and S4C) decreased and increased, respectively, within the Sound during these periods.

**Table 2:** Universal kriging results for the predicted mean salinity across 14 years (normal), mean salinity for three extremely wet years (wet) and mean salinity for three extremely dry years (dry) during both summer and winter seasons.

Season	N	Model	Nugget	Partial Sill	Total Sill	Theoretical Range (km)	Effective Range (km)
Summer							
Normal	227	Exponential	2.56	14.5	17.1	9.28	27.8
Wet	172	Exponential	4.14	16.3	20.4	18.7	56.0
Dry	184	Spherical	1.86	19.2	21.0	10.8	10.8
Winter							
Normal	219	Spherical	5.40	23.2	28.6	20.0	20.0
Wet	176	Gaussian	17.4	16.8	34.2	8.51	14.7
Dry	197	Exponential	0.88	30.2	31.1	6.10	18.3

N is the number of long-term stations used in each analysis. Nugget represents discontinuity at the origin due to microscale effects or measurement error. Total sill is the variance estimate. Theoretical and Effective ranges are the distances at which sampling stations are no longer spatially autocorrelated.

**Figure 3:**
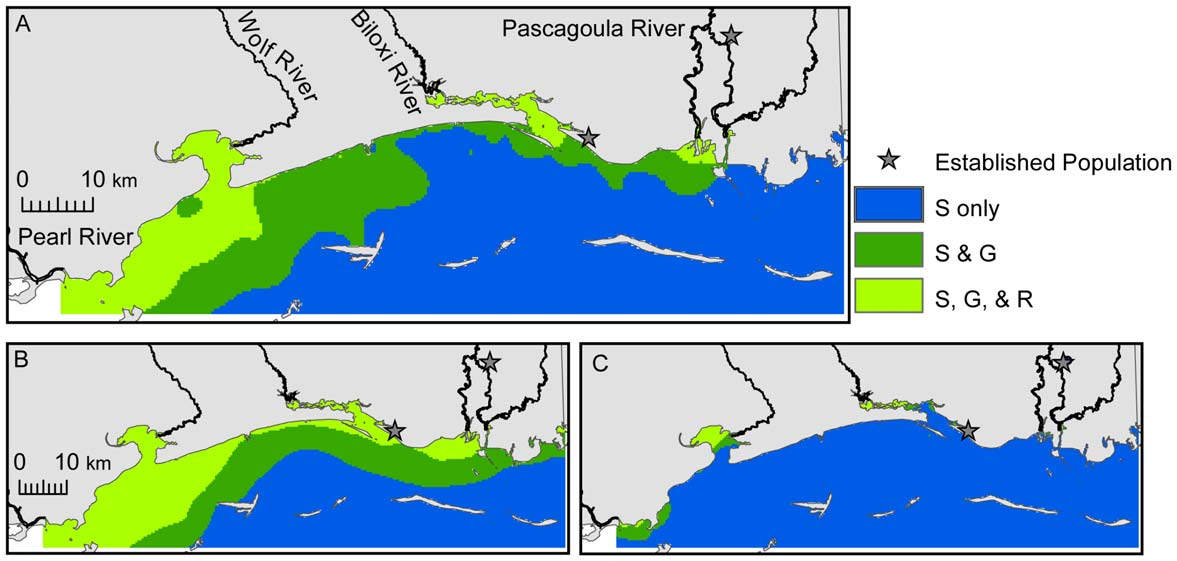
Areas of the Mississippi Sound with the highest probability of *Oreochromis niloticus* survival (S), growth (G), and reproduction (R) during summer months. A) normal years, B) wet years, and C) dry years.

**Figure 4:**
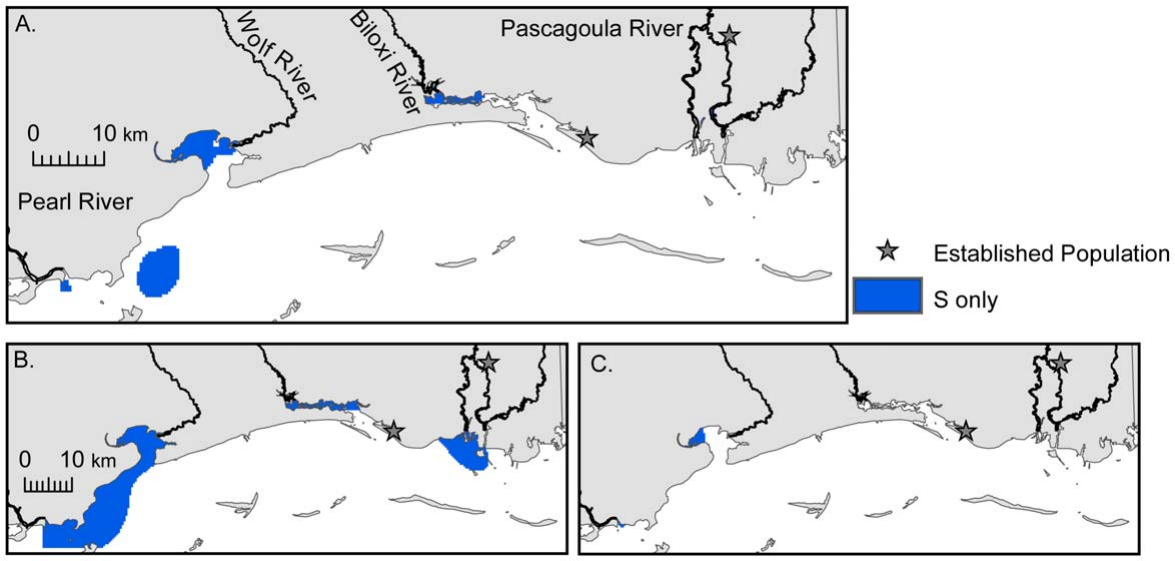
Areas of the Mississippi Sound with the highest probability of *Oreochromis niloticus* survival (S) during winter months. A) normal years, B) wet years, and C) dry years.

During normal summer salinity conditions, the probability of survival was high throughout the Sound (Figure S5A). However, the probabilities of positive growth (Figure S4B), reproduction (Figure S5C), and winter survival (Figure S6) decreased from inshore to offshore. Though Nile tilapia can survive throughout the Sound in the summer, growth and reproduction are limited to nearshore and low salinity habitats (Figure 3A), respectively. In the winter, survival is restricted to upper estuaries and rivers where salinity is typically <10 (Figure 4A). During extremely wet and dry years, the areal coverage of summer survival habitat did not change (Figures 3B, 3C; Table 3) relative to normal years. However, the areal coverage of growth and reproductive habitats increased by 2% and 47%, respectively, (Figures 3B, 3C; Table 3) for wet years. Conversely, growth and reproductive habitats decreased by 92% and 87%, respectively, during dry years due to increased salinity (Figures 3B, 3C; Table 3). Similarly, overwinter survival habitats increased by 368% during wet years (Figure 4B; Table 3) and decreased by 86% during extremely dry years (Figure 4C; Table 3).

**Table 3:** Areal coverage (km^2^) of survival, growth, and reproductive habitats for *Oreochromis niloticus* in the Mississippi Sound for normal salinity years, wet years, and dry years.

	Normal years		Wet years		Dry years	
Parameter	Summer	Winter	Summer	Winter	Summer	Winter
Survival	2.31	0.0763	2.31	0.281	2.31	0.0104
Growth	1.14		1.16		0.0946	
Reproduction	0.329		0.615		0.0438	

**All values are ×10^4^.**

**Figure 5:**
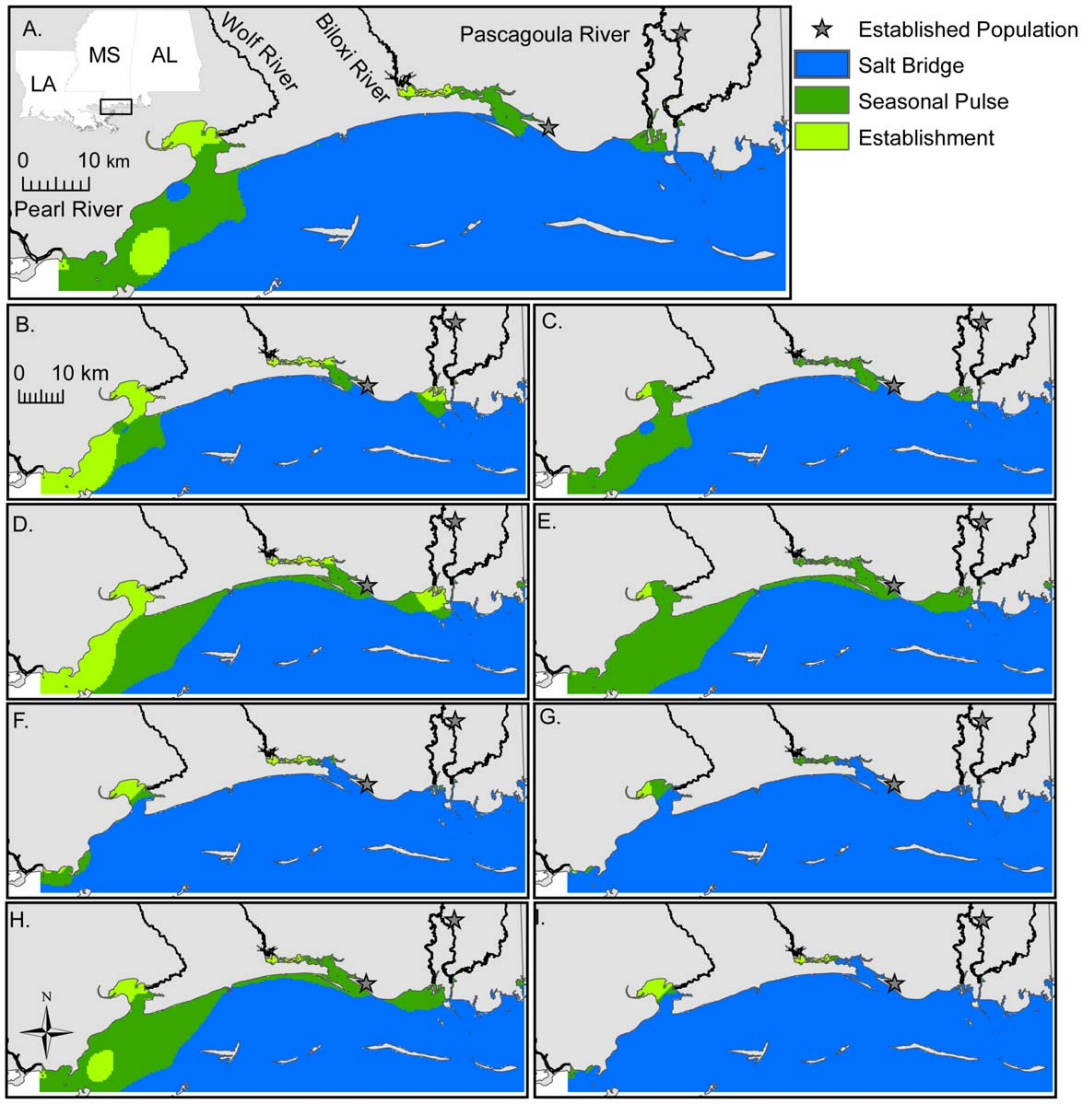
Projected areas of the Mississippi Sound that *Oreochromis niloticus* might use to establish a population (survive, grow, reproduce, and overwinter), pulse seasonally in abundance (survive, grow, reproduce, but not overwinter), or use as a salt bridge between adjacent freshwater systems (Survive and possibly grow, but reproduction and overwintering are unlikely). Each panel is for the following scenario: A) normal summer and normal winter, B) normal summer and wet winter, C) normal summer and dry winter, D) wet summer and wet winter, E) wet summer and dry winter, F) dry summer and wet winter, G) dry summer and dry winter, H) wet summer and normal winter, and I) dry summer and normal winter.

Given the physiological tolerances of Nile tilapia and the range of salinities commonly found in this region, the coastal waters of the Sound do not act as a barrier to dispersal. While seasonal pulses in abundance are likely to occur in the bays and estuaries, establishment of this species is likely limited to freshwater habitats and and low salinity habitats at the river mouths (Figure 5A). Further, under the different scenarios of climate variability (Figure 5B–I), the relative area of these different habitats will likely expand during extremely wet years (e.g., Figure 5D) and contract during extremely dry years (e.g., Figure 5G).

## Discussion

The growing effort to model the spread of invasive species [31]–[34] has highlighted the need for a proactive, predictive approach to invasive species management [35], [43]. The time lag between successive stages of the invasion process is not only unpredictable [44] but also hampers our ability to both monitor the spread and mitigate the impacts of biological invasions [45]. By integrating the empirical results of Schofield et al [22] with a predictive framework, we have expedited processes at the population level that are typically only observable over extended time scales, thus allowing us to proactively predict areas of the Sound that are susceptible to invasion by Nile tilapia. Our fundamental niche projections not only corroborate the ‘salt-bridging’ hypothesis discussed in Schofield et al. [22] but also highlight that nearshore, low-salinity waters, particularly in the western portion of the Sound, currently provide suitable conditions for establishment.

The present study also highlights the linkage between future precipitation patterns and the potential for Nile tilapia to spread throughout the region. For example, increased precipitation will likely facilitate the spread of Nile tilapia by increasing the areal coverage of habitats where they may establish. Conversely, decreased precipitation may limit the area available for Nile tilapia establishment; however, spread remains possible given that regional salinity patterns will fall within Nile tilapia's physiological limits for survival. It should be noted that our study conservatively approaches the implications of climate change on the spread of Nile tilapia by only considering changes in salinity and treating temperature as a static variable, particularly in the winter. Given the expectation that predicted increases in global temperature [46] will favor invasive species [41], [42], [47], warming regional temperature will likely have two major impacts on the spread of Nile tilapia. First, northward expansion in river systems becomes more likely as regional air and water temperature increases [48]. Secondly, areas that are not currently projected to promote establishment are likely to become suitable as physiological barriers breakdown during warmer winters.

To date, however, Nile tilapia has primarily been documented in freshwater habitats throughout the region and there have been three confirmed documentations directly in the Sound [49]. While this potentially reflects a discrepancy between fundamental and realized niches by overlooking key habitat characteristics (i.e., sediment type for bower (i.e., nest) formation), we suggest two probable explanations for the lack of observations within the Sound. First, sampling efforts targeting Nile tilapia have been biased towards freshwater habitats [7], [25] and routine fisheries-independent monitoring within the Sound uses sampling gear (e.g., long-lining, large mesh gillnets, trawling) that are not likely to capture Nile tilapia. Second, most invasive species exhibit an extended time lag between initial establishment and the onset of rapid population growth and spread [50]. Nile tilapia life history is characterized by low fecundity and high parental investment [51], [52]. Therefore, it may take several generations for populations to reach a level where local resources become limiting and thus warrant movement to new areas. However, there is wealth of evidence indicating that, as a whole, members of the family Cichlidae are fully capable of both moving through and establishing populations in low salinity, coastal waters both within their natural [53] and introduced ranges [54]–[57]. In Florida, for example, Mayan cichlid (*Cichlasoma urophthalmus*) established in freshwater systems are seeding ephemeral populations in estuarine habitats leading to a general northward expansion of the species into previously un-invaded systems since 1983 [55].

The potential for establishment of Nile Tilapia in the western Sound has much broader implications given the proximity to Louisiana's coastal habitats. Lake Pontchartrain, for example, is a large (1,839 km^2^), highly altered, shallow, low salinity estuary situated north of New Orleans, Louisiana that receives decreased freshwater inputs from the Mississippi River due to historical modification [58]. Proposed plans to restore freshwater input include periodic opening of flood control structures created by the U.S. Army Corps of Engineers [59], [60], which would reduce salinity in the basin by up to 40% [61]. The entrance to Lake Pontchartrain is <5 km from the mouth of the Pearl River and the system already supports the invasive Rio Grande cichlid (*Herichthys cyanoguttatus*) [57]. Therefore, current salinity patterns are not likely to differ from those in the western Sound. Increasing freshwater input into the basin would increase the probability of Nile tilapia establishment and provide them with a direct connection to adjacent systems, including the Mississippi River. However, restoring natural freshwater flows into Lake Pontchartrain could also aid the recovery of natural predators [62] that could act as a biological control for invasive Nile tilapia [63].

The ecological niche is conceptualized as all the biotic and abiotic factors that affect the expression of a species' distribution and can be further separated into the fundamental and realized niches [64]. Though the focus of this work is to project the fundamental niche of Nile tilapia using salinity and temperature, there are a multitude of biotic and abiotic factors that potentially shape the distribution and spread of this species in the northern GOM. Previous work showed that established Nile tilapia do not compete directly with native sunfishes (Family: Centrarchidae) for food in coastal Mississippi [65] and their aggressive behavior allows them to outcompete other native fishes for space [12]. The spectrum of potential predators of Nile tilapia in the northern GOM is similar to those in their natural range [66] and includes American alligator (*Alligator mississippiensis*), brown pelicans (*Pelecanus occidentalis*), and large piscivorous fishes such as alligator gar (*Atractosteus spatula*) and bull shark (*Carcharhinus leucas*). However, both alligator gar and bull shark abundances have declined in recent decades [62] and, though possible, it is unlikely that Nile tilapia would experience predation rates exceeding those of native fishes. However, the composition of both coastal [48] and freshwater [67] ichthyofaunal assemblages are expected to change with warming temperatures which may result in unpredictable species interactions.

Other abiotic factors that may be important drivers of Nile tilapia spread include dissolved oxygen, pH, depth and substrate type. Though bottom water hypoxia is a normal occurrence in the northern GOM [68], dissolved oxygen concentrations are generally within the optimal range for Nile tilapia [66]. Further, large-scale hypoxic zones associated with the Mississippi River occur in offshore waters outside of the depth preference for Nile tilapia [66]. The size and location of these hypoxic zones are driven principally by increased nutrient loads and the role of future climate change on these zones is unclear [69]. Conversely, while the pH of coastal draining rivers and backwater areas in the northern GOM [70] is within a tolerable range for Nile tilapia [71], the expectation is that pH will decrease due to ocean acidification [72], [73]. Nile tilapia are tolerant of a wide pH range but juvenile mortality is increased in acidic waters (pH<3.0) [71]. Temperature and salinity are the major metabolic modifiers for most tilapiine fishes [71] and, thus, we considered both to be the principal drivers of spread for invasive Nile tilapia in the northern GOM.

Once established, the ecological, evolutionary, and economic impacts of an invasive species can range from negligible to severe [74], [75]. However, there is a growing body of work showing that Nile tilapia can alter the function of aquatic systems through eutrophication [76], [77], altered trophic dynamics [12], and local extinction of native fish populations [66]. Generalizing the ecological impacts of a single invasive species remains a challenging task given the time required for the manifestation of such impacts [44] and lack of funding in this area [78].

### Ethics Statement

All animal work conducted in Schofield et al [22] and reproduced in this paper was done in accordance with the ‘Guidelines for the Use of Fishes in Research’ published by the American Society of Ichthyologists and Herpetologists (http://www.asih.ort/files/fish%20guidelines.doc) and approved by the U.S. Geological Survey, Southeast Ecological Science Center, Institutional Animal Care and Use Committee permit number USGS/FISC 2007-01.

## Methods

We constructed a predictive model based on the derived survival, growth, and reproduction data of Nile tilapia exposed to different combinations of salinity and temperature in an experimental setting (see Chronic salinity-tolerance experiment) [22]. Though a full recount of the experiment is beyond the scope of this paper and can be found in Schofield et al. [22], a brief discussion of the experimental design is warranted. Nile tilapia were housed in individual tanks and gradually acclimated (salinity of 5 per week) to target salinities (0 to 70 in increments of 10) at constant summer (30°C) and winter (14°C) temperatures, reflecting seasonal water conditions in coastal Mississippi. At the time of death or the end of the experiment fish were sacrificed by immersion in an ice water bath, weighed (±0.1 g), measured (±0.1 cm), and frozen. For each individual, survival was estimated with a Kaplan-Meier product limit estimator and both growth and spawning preparedness were quantified.

These data were converted to a binary response for logistic regression (see Step 1, Figure S1). Individuals surviving >65% of the total time at their target salinity were classified as ‘survived’ and growth was quantified as the change in body mass divided by the total number of experimental days (acclimation plus time at target salinity). Relative growth rates were converted to a binary response such that an individual exhibited either positive (net mass gain) or negative (net mass loss) growth during the experiment. For the reproductive measurements, we chose to restrict our analysis to female Nile tilapia because the maternal contributions to offspring can have a large impact on population dynamics and evolutionary plasticity of future generations [79], [80]. In Schofield et al.'s [22] summer experiment, all female Nile tilapia reared at a salinity ≤20 had GSI values ≥1.8 and produced large, vitellogenic oocytes while individuals reared in higher salinities had GSI values <1.8 and showed a marked reduction in vitellogenic oocyte production. Thus, an individual was deemed “spawning capable” or “spawning incapable” if its GSI value was ≥1.8 or <1.8, respectively. All Nile tilapia in the winter experiment exhibited negative growth and there was no evidence of reproductive development (i.e., GSI<0.75); thus, only survival probabilities were calculated.

We used the logistic regression function (Eq. 1) to model the survival, growth and reproduction probabilities of Nile tilapia from the summer experiment and the survival probabilities for the winter experiment separately:


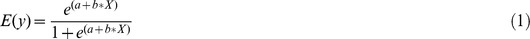


where *E*(y) is the probability of a positive outcome in survival, growth or reproduction, *a* and *b* are the intercept and slope in the linear link function, respectively, and *X* is the salinity covariate predictor. Due to both small sample size (summer survival, n = 94; growth, n = 76; reproduction, n = 38; winter survival, n = 70) and ‘quasi-complete’ separation of the experimental data (i.e., perfect correspondence between survival, positive growth, or reproductive capacity and salinity levels), finite parameter estimates did not exist using a GLM framework for logistic procedures [81]. As a result, we used a Firth-logistic regression (FLR) [82] to estimate survival, growth, and reproduction probabilities as a function of salinity for each season separately. The FLR uses a modified score function to split each original binary observation into “response” and “non-response” components, thus guaranteeing that each level of the predictor has some dichotomy of response variables and eliminating the problems associated with data separation [83]. It also uses penalized maximum likelihood (PML), carried out iteratively until parameter convergence, to estimate the logistic regression parameters, associated standard errors, and 95% confidence intervals [84]. The significance of each model parameter estimate was evaluated with the Wald score (WALD; *z*-statistic) and the likelihood ratio test (LRT; χ^2^) was used to assess goodness of fit by comparing the full model to the intercept only model. Nagelkerkes's *R* ^2^ was calculated for each model with the equation (Eq. 2):


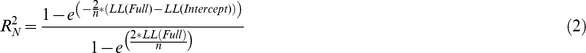


where *e* is the base of the natural logarithm, *LL*(*Full*) and *LL*(*Intercept*) are the penalized log-likelihood's associated with the full and intercept-only models, and *n* is the sample size [85]. All analyses were done using the logistf package in R [86] at a significance level of 0.05.

Seasonal salinity patterns in the Sound were examined from surface salinity and temperature data acquired from long-term data sets collected by various state and federal agencies in coastal Mississippi since 1973. Though each agency sampled at different temporal and spatial scales, we organized the data into a single relational database for continuously monitored stations. The database was checked for errors (e.g., out of range values) and then queried by temperature to generate salinities corresponding to Schofield et al.'s [22] summer (30±2.5°C) and winter (14±2.5°C) temperature conditions. For each season, we generated 3 data sets: 1) mean salinity across 14 years (1 January1992 through 15 March 2011) for both summer (227 stations) and winter (219 stations) conditions (Figure 1), hereafter normal salinity; 2) mean salinity for three extremely wet years during summer (1997, 2001, and 2003; Figure S1A, 1B; 172 stations) and winter (1998, 2004, 2009; S1C, 1D; 176 stations) conditions; and 3) mean salinity for three extremely dry years during summer (1996, 2000, 2006; Figure S1A, 1B; 184 stations) and winter (1999, 2000, 2007; S1C, 1D; 197 stations) conditions. River discharge data was attained from the US Geological Survey (USGS; http://waterdata.usgs.gov/nwis/rt) and wet and dry years were selected based on abnormally high or low mean discharge, respectively, during the summer and winter months for the Pearl, Wolf, Biloxi, and Pascagoula rivers (Figure S2) and maximized spatial coverage for each data set. The latter two data sets were used to examine how climate variability might impact the fundamental niche and potential spread of Nile tilapia.

Universal kriging was used to interpolate salinity between sampling stations in the Sound (4,792 km^2^) for each data set (see Step 2, Figure S1). Kriging is a group of geostatistical techniques that uses a set of linear regression routines to construct statistically optimal interpolation of a regionalized variable at unobserved locations (hereafter location) from spatially explicit samples (hereafter stations). Each interpolated value in a given location (0.16 km^2^ cell) is a weighted mean of the salinity at each sample (Figure 1) where the weights are based on the fitted semivariogram model derived from sampled values and the spatial configuration of the sampling stations. Due to an expected spatial trend for observed salinity (i.e., salinity decreased toward river mouths) within the Sound, we chose universal kriging. Universal kriging assumes the following model (Eq. 3):


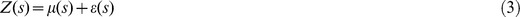


where *Z* is the interpolated salinity at location *s*, *μ* is the deterministic drift or trend modeled as a linear regression from geographic coordinates (WGS84 UTM 16 N projection), and *ε* is a stationary random variable that accounts for the spatial autocorrelation among sampling locations. We used the spherical, exponential, and Gaussian semivariogram models [87] to account for spatial autocorrelation and the model with the lowest sum of squares error was selected for salinity interpolation. For each semivariogram, we estimated the nugget, sill, and spatial range. The nugget represents a discontinuity at the origin due to microscale effects or measurement error [88]. The total sill is the sum of nugget effect and partial sill, and represents an estimate of the variance. The range denotes the distance at which the semivariogram reaches the sill, beyond which, there exists minimum spatial autocorrelation in the data of interest (e.g., salinity). For the exponential and Gaussian models, the semivariogram increases asymptotically toward its sill, so we calculated the effective range, defined as the distance at which the semivariance value achieves 95% of the sill. For the exponential and Gaussian models, the effective range is defined as the theorotical range multiplied by 3 and 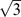, respectively. For the spherical model, the effective range is the theoretical range. Kriging was performed in R using gstat package, and the results were imported to ArcGIS 10.0 (ESRI 2009) for projecting the fundamental niche of Nile tilapia.

The derived predictive models (i.e., logistic regression) were applied to the whole Sound using interpolated salinity at each location as the covariate predictor (see Step 3, Figure S1). The simulation results were probabilities of survival, growth and reproduction in summer and probability of survival in winter. The locations with the probability of survival >0.95, probability of positive growth >0.70, and probability of reproduction >0.50 were classified as survival, growth, and reproduction habitats, respectively. Threshold values for each projection were selected at a level that realistically represented the results of Schofield et al. [22]. These were then projected for each season and used to compare changes in spatial coverage of those habitats between normal salinity years and extremely wet or dry years. The areal coverage of each habitat was calculated by multiplying the number of predicted cells by the spatial resolution of the salinity projections (0.16 km^2^). Further, we combined the seasonal habitat projections into a series of maps (see Step 4, Figure S1) in order to identify 1) areas of the Sound that promote survival, growth, and reproduction in the summer and where overwinter survival was most likely (the highest probability of establishment), 2) areas of the Sound where seasonal pulses in abundance might occur (i.e., survival, growth, and reproduction in the summer but not likely to overwinter), and 3) areas of the Sound that most likely function as a ‘salt-bridge’ between river systems (i.e., Nile tilapia can survive in the summer, but are not capable of spawning and overwintering is unlikely). A single projection indicating the highest probability of each area was generated for every seasonal combination of normal, wet, and dry years.

## Supporting Information

Figure S1 **Sketch diagram of the different steps and analyses performed in this study.** Long-term salinity and temperature data were complied from various state and federal agencies in coastal Mississippi since 1973.
(TIF)

Figure S2 **Mean (± Standard Error) discharge for the 4 major, coastal draining rivers in Mississippi.** Summer (A,B) and winter (C,D) river discharge for the Pascagoula and Pearl (A and C) and Wolf and Biloxi (B and D) rivers. Data acquired from real-time river monitoring. H and L indicate years used to generate salinity distributions for unseasonably wet and dry years, respectively.
(TIF)

Figure S3 **Predicted salinity during the summer (May thru September) for the Mississippi Sound.** A) normal years, B) wet years, and C) dry years.
(TIF)

Figure S4 **Predicted salinity during the winter (November thru February) for the Mississippi Sound during A) normal years, B) wet years, and C) dry years.**
(TIF)

Figure S5 **Projected probabilities of** ***Oreochromis niloticus*** **A) survival, B) growth, and C) reproduction in the Mississippi Sound during the summer.**
(TIF)

Figure S6 **Projected probabilities of** ***Oreochromis niloticus*** **survival in the Mississippi Sound during the winter.**
(TIF)
