# Supplementary figures and images for: Survival, Growth and Reproduction of Non-Native Nile Tilapia II: Fundamental Niche Projections and Invasion Potential in the Northern Gulf of Mexico
Source: PLoS One. 2012 Jul 27;7(7):e41580. doi: 10.1371/journal.pone.0041580 (PMC3407231; doi:10.1371/journal.pone.0041580)

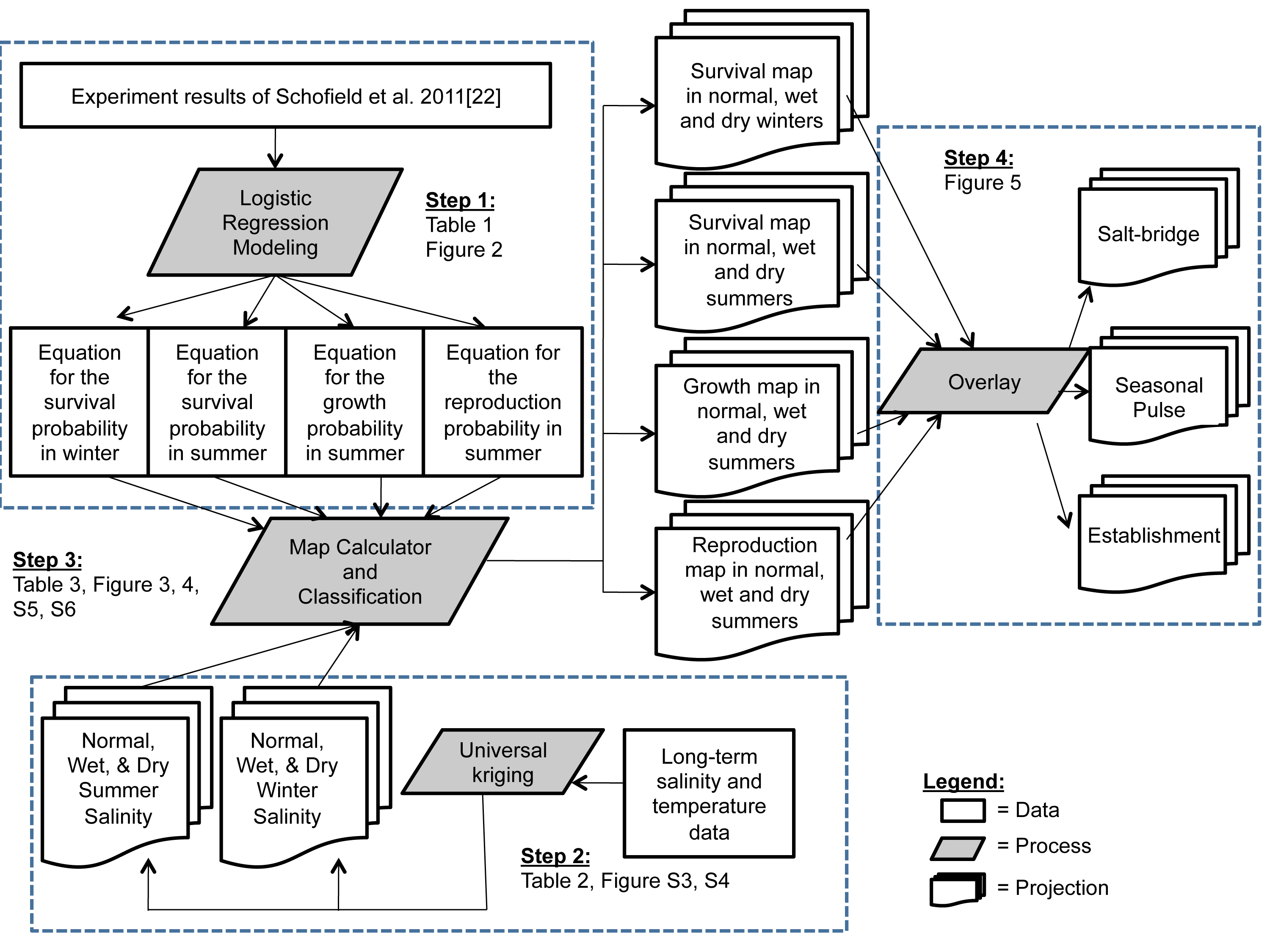

Supplement: Figure S1 — Sketch diagram of the different steps and analyses performed in this study. Long-term salinity and temperature data were complied from various state and federal agencies in coastal Mississippi since 1973. (TIF) [file pone.0041580.s001.tif]

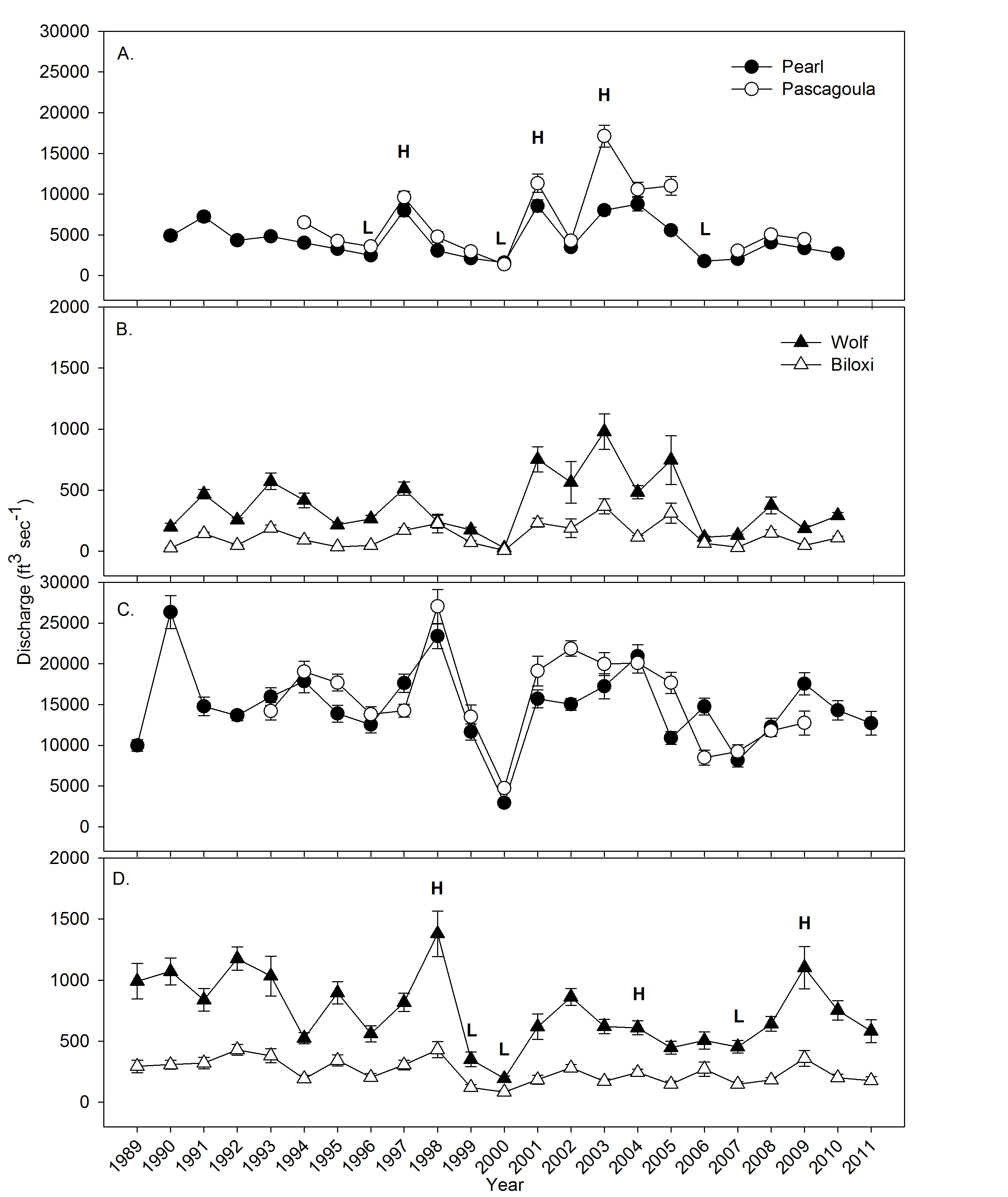

Supplement: Figure S2 — Mean (± Standard Error) discharge for the 4 major, coastal draining rivers in Mississippi. Summer (A,B) and winter (C,D) river discharge for the Pascagoula and Pearl (A and C) and Wolf and Biloxi (B and D) rivers. Data acquired from real-time river monitoring. H and L indicate years used to generate salinity distributions for unseasonably wet and dry years, respectively. (TIF) [file pone.0041580.s002.tif]

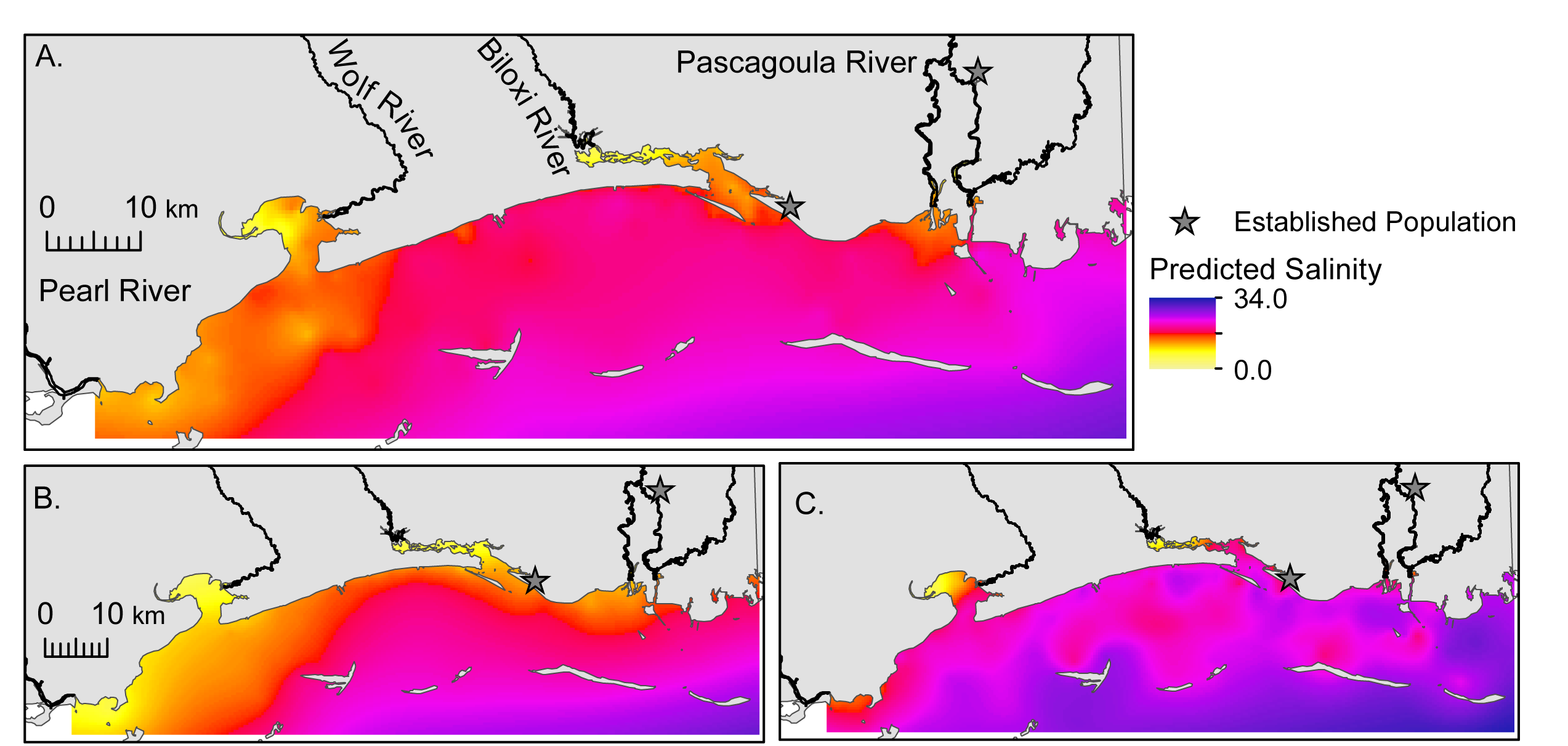

Supplement: Figure S3 — Predicted salinity during the summer (May thru September) for the Mississippi Sound. A) normal years, B) wet years, and C) dry years. (TIF) [file pone.0041580.s003.tif]

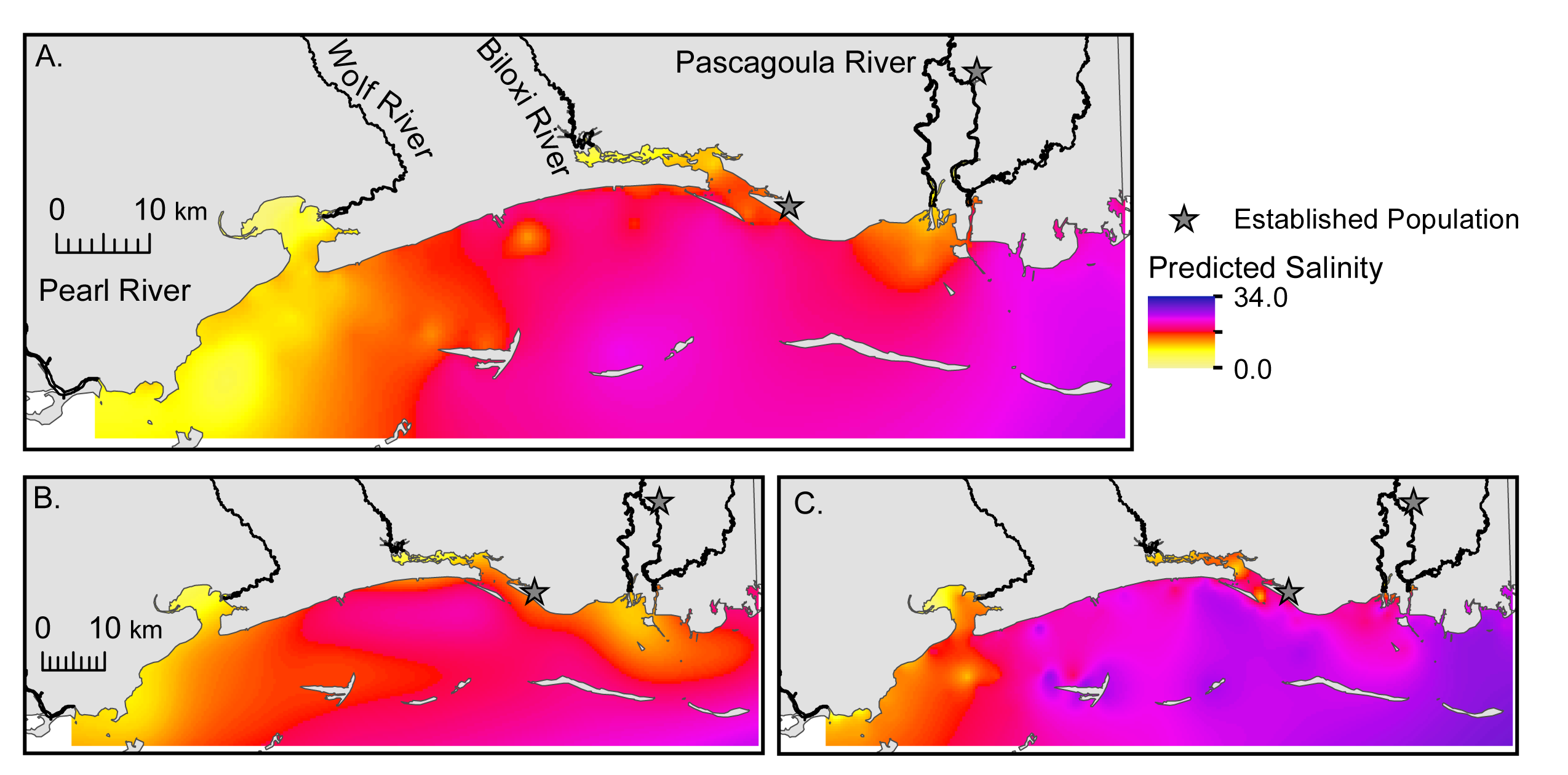

Supplement: Figure S4 — Predicted salinity during the winter (November thru February) for the Mississippi Sound during A) normal years, B) wet years, and C) dry years. (TIF) [file pone.0041580.s004.tif]

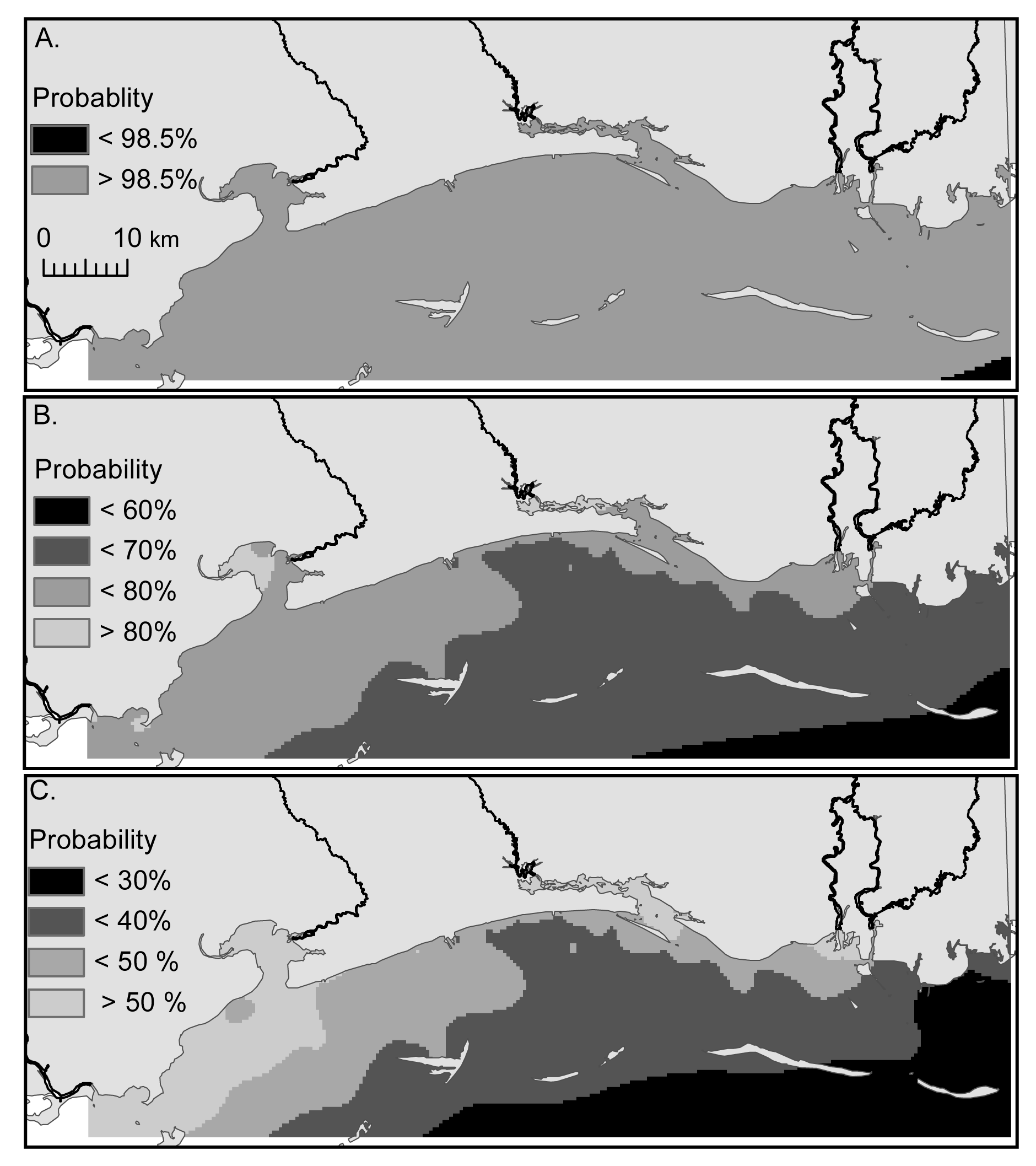

Supplement: Figure S5 — Projected probabilities of Oreochromis niloticus A) survival, B) growth, and C) reproduction in the Mississippi Sound during the summer. (TIF) [file pone.0041580.s005.tif]

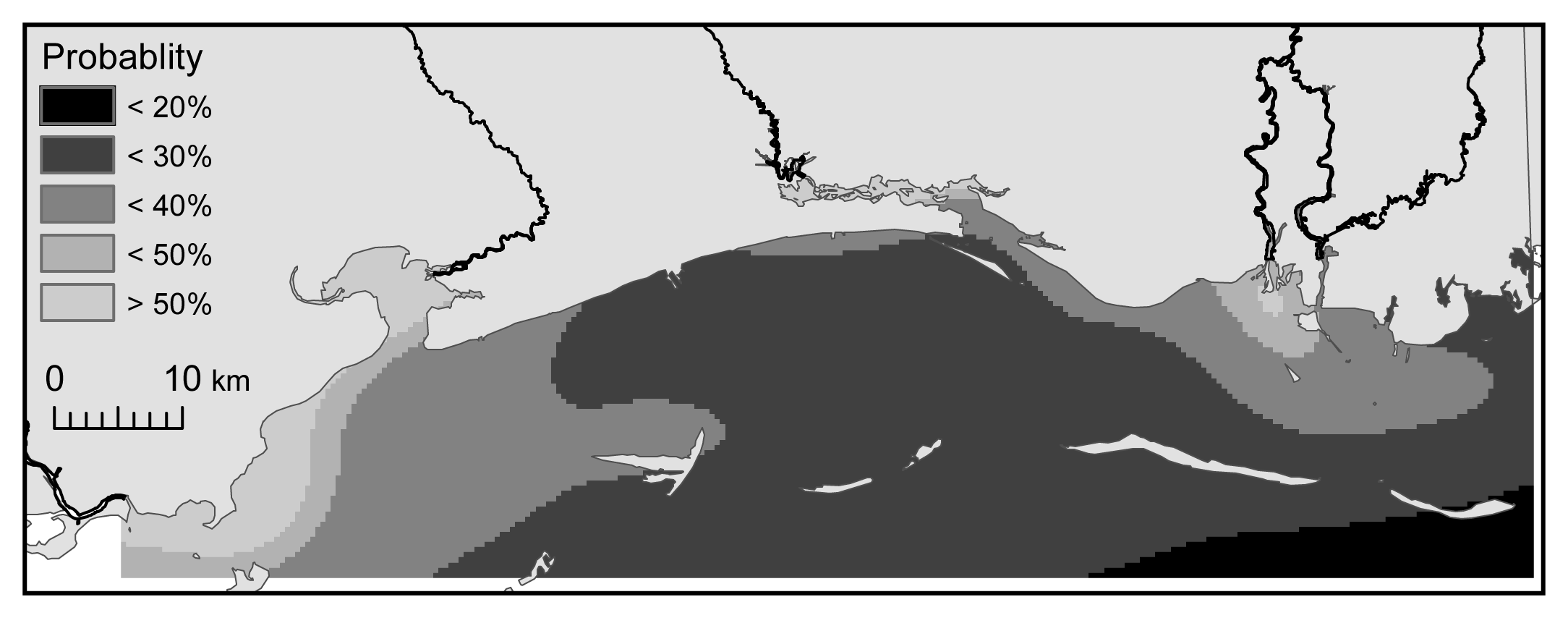

Supplement: Figure S6 — Projected probabilities of Oreochromis niloticus survival in the Mississippi Sound during the winter. (TIF) [file pone.0041580.s006.tif]
